# Non-Uniform Bioaccumulation of Lead and Arsenic in Two Remote Regions of the Human Heart’s Left Ventricle: A Post-Mortem Study

**DOI:** 10.3390/biom13081232

**Published:** 2023-08-10

**Authors:** Ana Cirovic, Orish E. Orisakwe, Aleksandar Cirovic, Jovan Jevtic, Danijela Tasic, Nebojsa Tasic

**Affiliations:** 1 Faculty of Medicine, Institute of Anatomy, University of Belgrade, Dr Subotica 4/2, 11000 Belgrade, Serbia; ana.cirovic@med.bg.ac.rs; 2 African Centre of Excellence for Public Health and Toxicological Research (ACE-PUTOR), University of Port Harcourt, PMB, Choba, Port Harcourt 5323, Nigeria; orish.orisakwe@uniport.edu.ng; 3 Faculty of Medicine, Institute of Pathology, University of Belgrade, Dr Subotica 1, 11000 Belgrade, Serbia; lordstark90@gmail.com; 4 Institute for Cardiovascular Diseases Dedinje, 5 Heroja Milana Tepica Street, 11000 Belgrade, Serbia; dtasic74@yahoo.com (D.T.); nebtasa@yahoo.com (N.T.); 5 Faculty of Medicine, University of Banja Luka, Banja Luka 74278, Bosnia and Herzegovina; 6 Faculty of Medicine, University of Belgrade, Dr Subotica 8, 11000 Belgrade, Serbia

**Keywords:** arsenic, lead, left ventricle, post-mortem

## Abstract

The extent of heavy-metal-induced cardiotoxicity is proportional to the levels of metal bioaccumulation, and it was previously assumed that heavy metals accumulate uniformly in the myocardium. Therefore, the aim of this study was to investigate concentrations of metals and metalloids in two distant regions of the left ventricle (LV), the base of the LV, and apex of the LV using inductively coupled plasma mass spectrometry (ICP-MS). We also examined the potential correlation between metal levels and the thickness of the interventricular septum in twenty LV specimens (ten from the base of LV and ten from the apex of LV) from 10 individuals (mean age 75 ± 6 years). We found significantly higher concentrations of arsenic and lead in the LV apex compared to the base of the LV. We also found a positive correlation between the concentrations of arsenic in the myocardium of LV and the thickness of the interventricular septum. Our results indicate that arsenic and lead accumulate to a higher extent in the apex of the LV compared to the base of the LV. Therefore, future studies designed to measure levels of metals in heart muscle should consider non-uniform accumulation of metals in the myocardium.

## 1. Introduction

Cardiomyocytes are highly sensitive to toxicity induced by heavy metals. Most studies conducted so far have shown that the adverse effects of toxic metals, such as cadmium (Cd) and lead (Pb), are dose-dependent [1,2] and can lead to the deterioration of cardiomyocytes function [2]. Many authors have also demonstrated that the levels of Cd and Pb can vary significantly between individuals and different organs within the same person [3,4]. Egger et al. found that the levels of Cd varied by more than 4500-fold among various human tissues [4]; therefore, it is reasonable to speculate that Cd and other heavy metals could accumulate non-uniformly within the same organ.

Humans are exposed to various heavy metals on a daily basis through their daily routine. The intestinal tract is the main route of heavy metal entry into the body as they are often present in various foodstuffs [5,6]. The respiratory tract represents a significant body entrance route for heavy metals in individuals who smoke [7], and smokers have been found to have higher blood levels of Pb and Cd in some studies while others have reported elevated concentrations of thallium (Tl), arsenic (As), manganese (Mn), and copper (Cu) in the serum of smokers [8]. The harmful effects of heavy metals on the heart muscle are indisputable. A prospective study conducted in Sweden with a 17-year follow-up period showed that increased blood levels of Cd are linked with the appearance of heart failure [9]. Exposure to Pb represents a risk factor for cardiovascular mortality; a large prospective study with a follow-up period of 19.3 years which included 18,825 adults aged 20 years and older revealed that a rise in blood Pb levels from 1.0 μg/dL to 6.7 μg/dL (which spans from the 10th to the 90th percentiles) was linked to a marked increase in all-cause mortality, cardiovascular disease mortality, and mortality related to ischemic heart disease [9]. Moderate exposure to As in drinking water was found to be significantly linked to a higher risk of cardiovascular disease among residents who were exposed to it [10].

A study conducted by Ali et al. showed that male and female rabbits aged 1 month, weighing between 1 and 1.5 kg, that were orally fed with Cd-chloride at a concentration of 1.5 μg/g for a period of 28 days, exhibited a significant increase in serum levels of troponin T and creatine kinase (markers of myocardial injury) compared to the control group [11]. Li et al. reported that Pb induced apoptosis of rat cardiomyocytes (H9c2 cells) in a dose-dependent relationship [1]. It is important to note that some metals, such as iron (Fe) or Cu, that are beneficial for human health, could express their toxicity if found in high concentrations [12,13]. On the other hand, certain elements, such as Zn, can decrease cellular antioxidant mechanisms when present in low concentrations [14]. Additionally, low cellular Zn concentration may enhance the toxic effects of heavy metals, as reduced Zn levels are associated with decreased levels of metallothionein—a protein that binds to heavy metals and mitigates their toxic effects [14].

The left ventricle plays a crucial role in pumping blood to the systemic circulation, and its proper function is essential for maintaining normal body function. A decrease in the functioning of 20 percent of the left ventricle myocardium can potentially result in heart failure. Hence, it becomes crucial to predict the levels of heavy metals in the heart during examinations, as higher concentrations have been associated with reduced heart function. However, the number of studies examining this matter remains limited, and so far, only one study has explored the correlation between blood metal levels and some aspects of heart geometry such as the thickness of the interventricular septum [15].

Recently, Cirovic et al. provided a detailed description of the mechanisms involved in the increased body accumulation of cadmium in individuals with chronic obstructive pulmonary disease (COPD) and iron deficiency anemia (IDA). In brief, the divalent metal transporter 1 (DMT1) and transferrin receptor 1 (TfR1) are hypoxia-inducible genes [16,17]. In the case of systemic hypoxia (COPD and IDA), DMT1 becomes overexpressed in the intestine, resulting in the enhanced absorption of all divalent cations, including Pb and Cd.

On the other hand, atherosclerosis is a process that typically begins around the age of 20 and affects almost all arteries in the human body, including those in the heart. Atherosclerosis can result in the occurrence of local hypoxia distal to the atherosclerotic plaque. It is reasonable to speculate that more distant regions of the heart muscle, e.g., those closely related to the anterior interventricular branch of the left coronary artery (e.g., the apex of the left ventricle), experience more hypoxic conditions compared to the more proximal myocardium. This is because atherosclerosis can cause various degrees of stenosis along the vessel, which may not result in severe hypoxia but can lead to relative hypoxia compared to the more proximal parts of the myocardium.

Consequently, the myocardium of the left ventricular apex could express DMT1 and transferrin receptor 1 (TfR1) at a higher density and uptake heavy metals from the blood to a greater extent, since the majority of metals are transported while bound to transferrin, albumin, and other proteins [18,19].

Given that heavy metals, such as cadmium or arsenic, are almost 100-fold more toxic to cardiomyocytes than to other cell types [20], and that the adverse effects of most toxic metals are dose-dependent [21], the aim of this study was as follows: to investigate whether metals, including heavy metals, accumulate uniformly in the two remote regions of the left ventricle, and consequently whether measuring metals levels in only one region of the left ventricle or heart is justified; and ii) to determine whether there is a correlation between metal concentrations and interventricular septum thickness (IVST). Furthermore, the rationale for selecting these two regions of the left ventricle (LV) can be attributed to their distinct functional roles. Unlike the base of the LV, the LV apex is known for its heightened muscular activity and is widely acknowledged as the actively contracting section of the heart. Hence, it was deemed imperative to compare these two regions in the context of the toxic and non-toxic elements analysis.

## 2. Materials and Methods

### 2.1. Sample Collection

**Figure 1:**
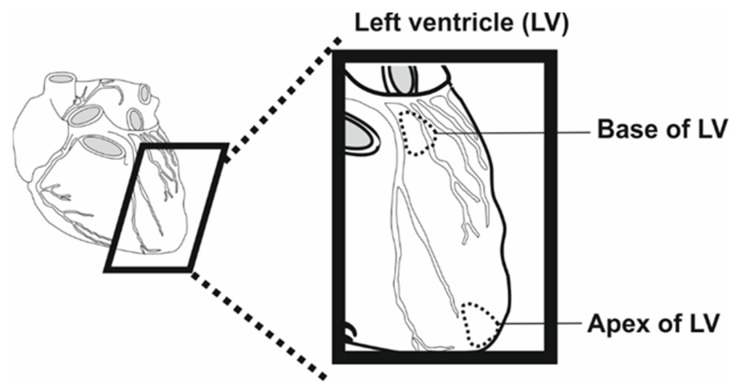
Schematic representation of sampling sites.

We collected 20 human left ventricle (LV) specimens from 10 donors at the Institute of Anatomy at Belgrade University. Ten samples of the heart muscle were taken from the LV apex, and the remaining ten were taken from the base of the left ventricle (Figure 1). The specimens were obtained during routine dissection and had dimensions of approximately 5 × 5 × 5 mm. After extraction, the samples were stored at −20 °C. The individuals whose bodies were used for the study had died due to traumatic injuries or aging-associated diseases, including hepatic disease, and had signed informed consent forms prior to their deaths for the use of their bodies for scientific and educational purposes by the Institute of Anatomy, University of Belgrade. The institutional Ethics Committee granted approval for the collection of the samples (17/IV-29).

### 2.2. Inductively Coupled Plasma Mass Spectrometry (ICP-MS) Measurement

After sample extraction from cadavers and storage, they underwent thawing. The wet sample tissue (approx. 0.5 g) was digested in a Teflon vessel with 5 mL of 67% HNO_3_ (Trace Metal Grade, Fisher Scientific, Bishop, UK) and 1.5 mL of 30% H_2_O_2_ (analytical grade, Sigma-Aldrich, St. Louis, MA, USA) for microwave digestion.

Microwave digestion was performed using a Start D microwave (Milestone, Sorisole, Italy) with a program that consisted of three steps: heating from to 180 °C in 300 s, holding at 180 °C for 10 min, and venting for 20 min. After the temperature dropped, the digested sample solutions were quantitatively transferred to disposable flasks and diluted to 100 mL using deionized water produced by a water purification system (Purelab DV35, ELGA, Buckinghamshire, UK).

An inductively coupled plasma mass spectrometry (ICP-MS) analysis was performed for magnesium (Mg), calcium (Ca), cadmium (Cd), lead (Pb), iron (Fe), zinc (Zn), copper (Cu), manganese (Mn), nickel (Ni), mercury (Hg), and arsenic (As) using the iCap Q mass spectrometer (Thermo Scientific, Bremen, Germany) (Table 1). The torch position, ion optics, and detector settings were adjusted daily with a tuning solution (Tune B, Thermo Scientific) to optimize mechanical and electrical parameters and minimize interference. The basic operating conditions of the instrument were as follows: RF power (1550 W); cooling gas flow (14 L/min); nebulizer flow (1 L/min); collision gas flow (He, 1 mL/min); operating mode (Kinetic Energy Discrimination KED); and dwell time (100 ms). The KED mode was used during analyzing to eliminate errors in the determination of iron that may arise from the Ar-O, which have the same mass as iron.

Standard stock solutions containing 1000 mg/L of each element (Fe, Zn, Cu, Mn, Ni, Na, K, Mg, Ca, Cd, Pb, Hg, and As) were purchased from CPAchem (Bogomilovo, Bulgaria) and used to prepare five-point calibration curves (including zero). A multielement internal standard (_6_Li, _45_Sc, _71_Ga, _89_Y, _209_Bi) was introduced online through an additional line via the peristaltic pump, covering a wide mass range. All solutions (standards, internal standards, and samples) were prepared in 2% nitric acid.

The analytical method was validated according to the Guidelines for Single Laboratory Validation (SLV) of the Chemical Methods for Metals in Food (AOAC) and accredited to ISO 17025. The quality of the analytical process was verified by analyzing certified reference materials NIST 1577c (bovine liver, Gaithersburg, MD, USA) and ERM-BB384 (lyophilized pork muscle, ERM, Geel, Belgium), which were prepared as samples using microwave digestion. The measured concentrations for all isotopes were within the range of certified values.

ICP-MS is a commonly used method for measuring metals in studies that have examined human tissues and fluids, such as urine or blood [22,23].

### 2.3. Measurement of LV Wall Thickness

We measured the thickness of the interventricular septum (IVST) close to the heart apex individually using vernier calipers. The measurement was performed by two researchers separately and mean values were included for statistical analyzes.

### 2.4. Statistical Analysis

The Kolmogorov–Smirnov test was used to verify that all the measured parameters complied with the normal distribution. The t-test or Mann–Whitney U test was used to check for differences mean concentrations of metals between the two examined sights of the left ventricle (apex and base of the LV). The Pearson (or Spearman) correlation test was performed to assess link between the metals concentrations with age and with interventricular septum thickness. All the analyses were performed two-tailed in SPSS software ver. 15 at the significance level of 0.05.

## 3. Results

**Figure 2:**
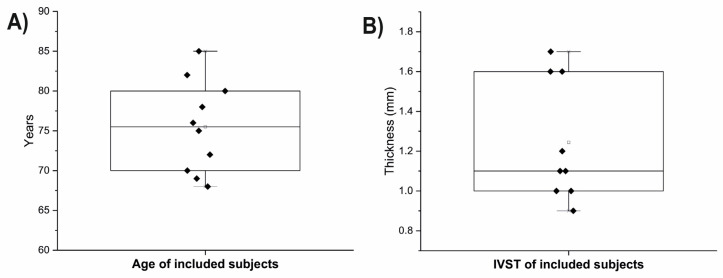
Age (**A**) and IVST (**B**) in examined individuals.

In this study, we included 10 individuals with an average age of 75 ± 6 years and measured the thickness of the interventricular septum, which was found to be 1.25 ± 0.3 centimeters (Figure 2). The sample consisted of five male and five female individuals.

**Figure 3:**
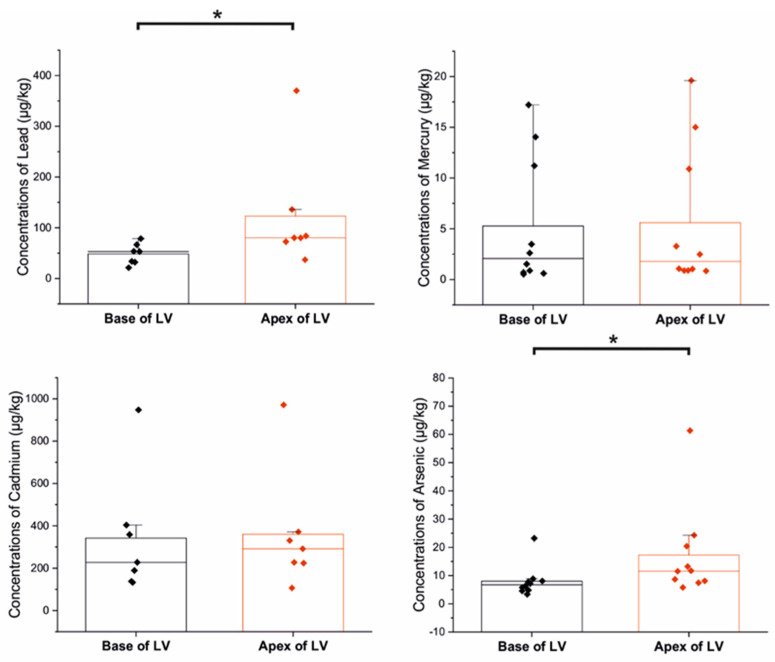
Concentrations of toxic metal(oid)s between examined sites (µg/kg); * *p* < 0.05.

**Figure 4:**
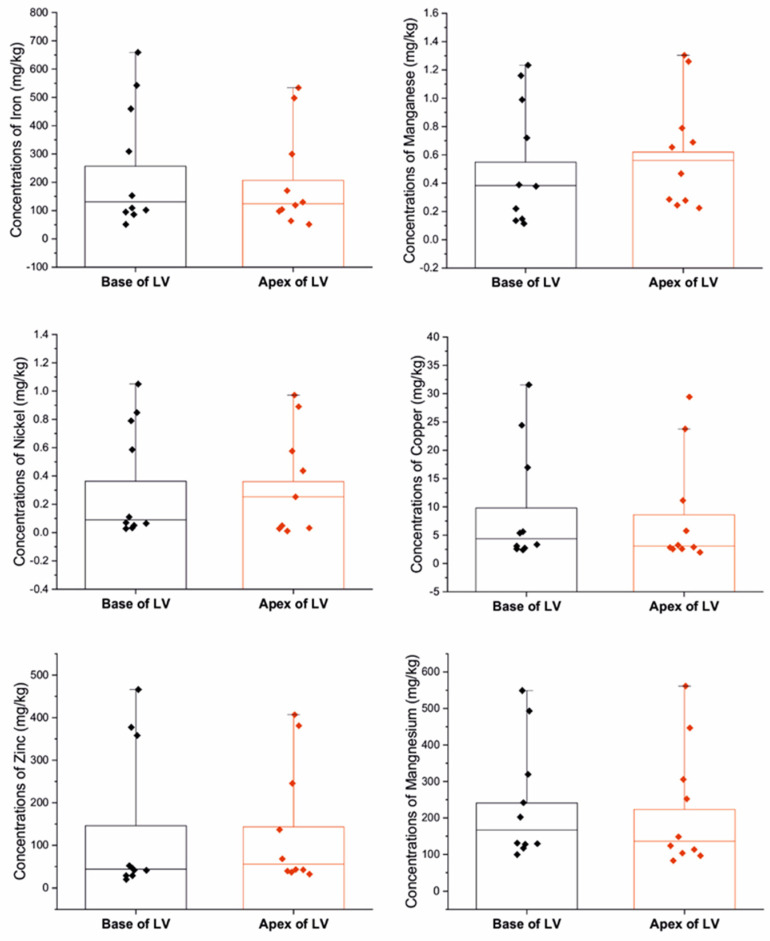
Concentrations of metals between examined sites (mg/kg); * *p* < 0.05.

We observed significantly higher concentrations of arsenic at the LV apex compared to the base of the LV (*p* = 0.0147) and higher levels of lead at the LV apex than at the base of the LV (*p* = 0.0111) (Figure 3). However, we did not observe any significant variation in the concentrations of other elements (calcium, cadmium, iron, mercury, magnesium, manganese, nickel, zinc, copper) between the two examined sites (*p* = 0.805, *p* = 0.902, *p* = 0.853, *p* = 0.684, *p* = 0.529, *p* = 0.436, *p* = 0.436, *p* = 0.579, *p* = 0.684) (Figure 4).

### Correlations between an Age and IVST with Metals Concentrations

We also investigated whether age or thickness of the interventricular septum (IVST) influenced the accumulation of metals in the LV myocardium independent of the site differences. We used the mean values of the two examined sites for this analysis. We did not find any significant correlation between age and the concentrations of arsenic, cadmium, copper, iron, magnesium, manganese, nickel, zinc, and lead (*p* = 0.302, *p* = 0.595, *p* = 0.556, *p* = 0.110, *p* = 0.354, *p* = 0.369, *p* = 0.354, *p* = 0.302, and *p* = 0.302). However, we found a significant negative correlation between Hg concentrations and age (r = −0.929, *p* = 0.007). Furthermore, we observed a significant positive correlation between the concentrations of arsenic in the LV and the thickness of the interventricular septum (r = 0.81, *p* = 0.008). However, we did not observe any significant correlation between the thickness of the interventricular septum and the concentrations of other elements (cadmium, iron, mercury, magnesium, manganese, nickel, zinc, lead, copper, and calcium) (*p* = 0.154, *p* = 0.795, *p* = 0.648, *p* = 0.526, *p* = 0.09, *p* = 0.291, *p* = 0.440, *p* = 0.305, *p* = 0.440, *p* = 0.897).

## 4. Discussion

### 4.1. Arsenic and Lead-Induced Cardiotoxicity

In a systematic review (with over 20 included papers) conducted by Moon et al., it was shown that individuals living in high-arsenic-exposure areas are at greater risk of developing coronary artery disease [24,25]. Pichler et al. conducted a population-based prospective cohort study in which they measured urine As levels and performed transthoracic echocardiography geometric measurements of the left ventricle. They found that the thickness of the interventricular septum and posterior wall of the LV correlated with As exposure at study baseline. Moreover, As exposure was associated with LV hypertrophy [15]. Zhao et al. conducted a meta-analysis that included 27 articles, demonstrating that arsenic exposure is linked with hypertension. Systematic reviews included in this meta-analysis indicated an association between chronic arsenic exposure and high systolic blood pressure [26]. In an analysis of NHANES 2003–2014 subjects (4990 participants were included in this study), urinary total arsenic concentrations were positively and significantly associated with heart disease mortality [25,27].

Mana et al. divided 24 adult male albino mice into four groups, where group 1 consisted of controls, group 2 was treated with NaAsO_2_ at a dose of 10 mg/kg per os for 2 days, group 3 was exposed to the same doses of NaAsO_2_ and co-treated with arjunolic acid, while mice of group 4 were co-treated with NaAsO_2_ and vitamin C. The authors performed biochemical and histological examination of mice hearts at the end of the experiment [27]. The authors reported that mice treated with NaAsO_2_ had attenuated activity of the following antioxidants: superoxide dismutase (SOD), catalase, glutathione-S-transferase, glutathione reductase, and glutathione peroxidase. Furthermore, NaAsO_2_ exposed animals had increased concentrations of malondialdehyde (MDA), which points to increased oxidative stress in the myocardium. In addition, mice treated with NaAsO_2_ showed altered histological parameters of the myocardium. Adil et al. demonstrated that adult male Sprague-Dawley rats exposed to sodium arsenite (5 mg/kg, p.o.) and treated with distilled water (5 mL/kg, p.o.) for 28 days experienced a decrease in heart rate coupled with a significant increase in QRS, QT, QTc, and RR intervals when compared to the control group [28]. Additionally, Adil et al. also showed that NaAsO_2_-treated rats had significantly higher serum levels of LDH, creatine kinase-myocardial band (CK-MB), aspartate aminotransferase (AST), alanine transaminase (ALT), and alkaline phosphatase (ALP) coupled with lower activity of SOD, reduced glutathione (GSH), and increased concentrations of MDA and NO (indicators of oxidative stress). Results of another in vivo study which included male Sprague-Dawley rats also indicated increased serum levels of CK and LDH in rats treated with arsenic trioxide with intraperitoneal injection for 10 days. Apart from obtaining increased markers of oxidative stress (MDA) and decreased concentrations of antioxidants such as SOD, glutathione peroxidase (GSH-Px), and catalase (CAT) in hearts of rats treated with As_2_O_3_, Zhao et al. [29] also found increased levels of pro-inflammatory cytokines (IL-1, IL-6, and TNF-α) in heart tissue. Arsenic also manifests pro-apoptotic effects by promoting activity of caspase-3 enzyme [28] and apoptotic genes, such as BAX and PUMA. Wang et al. and Li et al. examined the effects of Pb exposure in H9c2 cells (embryonic BD1X rat heart cells) and showed that exposure to lead led to increased release of cardiac enzymes such as LDH, AST, and CK-MB and deteriorated expression of Connexin 43 [1,2] which is important for cell-to-cell communication between neighboring cardiomyocytes.

We found that the greater the thickness of the interventricular septum, the higher the accumulation of arsenic within the myocardium. Our findings are in agreement with previously conducted studies, but with greater precision, as we directly measured both arsenic in tissue of LV and myocardial thickness. In contrast, Pichler et al. measured arsenic in blood, which may not fully reflect the concentration of arsenic in the myocardium. Therefore, increased thickness of the myocardium, obtained during routine echocardiographic examination, could potentially alert cardiologists about potentially increased accumulation of metals in the left ventricle; however, similar studies that include individuals with cardiological disorders and larger sample size should be conducted to confirm our findings. Decreased levels of mercury in aged individuals are also in agreement with previous studies [30].

As we discussed, As is toxic to certain selenoproteins (e.g., GSH-Px) [27]. Furthermore, it appears that heavy metals also have a toxic effect on contractile proteins, such as Troponins. Not only is Troponin affected by heavy metal toxicity, but the function of other proteins in the contractile myocardium apparatus may also be affected. Klinova et al. conducted an in vivo study to examine the cardiotoxic effect of Pb acetate. They administered a dose of 11 mg/kg body weight intraperitoneally over a 6-week period, three times a week, and discovered a significant reduction in the maximal velocity of reconstituted thin filament sliding over rat myosin under lead intoxication [31]. Gerzan et al. exposed male rats to intraperitoneal doses of 12.5 mg of Pb per kg body mass and 6.01 mg of Pb per kg body mass over a five-week period. Their study revealed that both doses interfered with optimal actin-myosin interaction. Specifically, Pb decreased the velocity of sliding between filaments in both the atria and ventricles [32].

As and Pb are highly cardiotoxic, and their adverse effects are dose-dependent. This further suggests that higher doses of these metals are linked to more deleterious effects on the heart muscle. The reason why the apex of the left ventricle “attracts” heavy metals to a greater extent remains to be investigated in the future. All in all, discussed papers unequivocally highlighted the biochemical aspects of adverse effects caused by heavy metals exposure on cardiomyocytes. The gradual accumulation of heavy metals, specifically As and Pb in our case, may lead to significant damage to cardiomyocytes. This damage can ultimately lead to the destruction of cardiomyocytes, which may trigger fatal arrhythmias. The release of intracellular potassium into the myocardial interstitium disrupts ventricular depolarization, causing delays and disorganization. We speculate that these effects could potentially contribute to the occurrence of sudden cardiac death (SCD). However, it is important to note that SCD is a multifactorial event with various contributing factors, and the role of heavy metals in its occurrence requires further confirmation through future studies.

Becker et al. conducted an interesting study where they practically mapped metals in the heart muscle using an in vivo model. The authors discovered that the distribution of Zn, Cu, Fe, and Mn within the myocardium is highly heterogeneous [33]. In contrast to our study, Becker et al. analyzed the entire heart using laser ablation inductively coupled plasma mass spectrometry (LA–ICP–MS). This technique provided them with precise data on metals’ bioaccumulation in both physiological and pathological conditions. Furthermore, LA–ICP–MS allows for the evaluation of multiple organ systems, including not only the heart but also the central nervous system [34,35].

### 4.2. Suggestions for a Future Measurements

The majority of studies that directly measured various metals in heart specimens post-mortem only took one sample of heart tissue [4,36,37,38]. The baseline assumption of all these studies is that metals uniformly accumulate within one organ, the heart in this case. However, since we found that concentrations of some metals may vary between two sites of the left ventricle, future studies that aim to measure metals in heart specimens should include at least two distant sites. Measuring only one site could limit the evaluation of the actual situation, e.g., concentrations of lead were twofold higher in the apex of the left ventricle compared to the base, and therefore sampling only one site of the left ventricle cannot be representative of the entire ventricle or organ (heart).

The present study has some limitations, including a limited sample size and a lack of information on the smoking status of subjects prior to death and other demographics. However, the absence of socioeconomic data may not have affected the obtained result when comparing the two sites of the left ventricle since they were exposed to the same levels of metals from the blood. Therefore, it can be concluded that intrinsic factors led to a higher accumulation of arsenic and lead in the apical myocardium of the left ventricle. Moreover, we expect that future studies, which will further examine this issue, will address the factors that contribute to the increased bioaccumulation of Pb and As in the heart apex. Additionally, these studies will investigate why these two metals are specifically accumulated.

## 5. Conclusions

As and Pb accumulation in the myocardium of the left ventricle is not uniform. The apex of the heart had higher concentrations of arsenic and lead. Levels of arsenic in the left ventricular myocardium positively correlated with the thickness of the interventricular septum.

## Figures and Tables

**Table 1:** RSDr—relative standard deviation of reproducibility; Samples with elements concentrations above the upper range were diluted appropriately.

Elements	Range	LOD	LOQ	RSDr	Recovery
	µg kg^−1^	µg kg^−1^	µg kg^−1^	%	%
_75_As	4–100	1.2	4	6.57	95.3
_111_Cd	1–100	0.4	1	8.99	100.4
_207_Pb	4–100	2	3.8	6.65	101.1
_202_Hg	1–100	0.3	0.9	6.90	96.0
	mg kg^−1^	mg kg^−1^	mg kg^−1^	%	%
_63_Cu	1–50	0.022	0.066	6.26	101.7
_57_Fe	5–300	0.08	0.23	4.71	96.6
_66_Zn	2–100	0.124	0.372	10.52	95.6
_55_Mn	0.1–5	0.004	0.011	4.47	102.0
_60_Ni	0.1–5	0.050	0.145	9.19	102.4
_44_Ca	10–500	3.08	9.24	3.64	99.6
_24_Mg	10–500	0.13	0.40	3.03	98.2

## Data Availability

Data available upon reasonable request.
